# Risk factors of striae gravidarum in Chinese primiparous women

**DOI:** 10.1371/journal.pone.0198720

**Published:** 2018-06-21

**Authors:** Liping Liu, Jianling Huang, Ying Wang, Yumei Li

**Affiliations:** 1 Department of Dermatology, Affiliated Hospital of Jiangsu University, Zhenjiang, Jiangsu, China; 2 Regeneration Medicine Research Center, Affiliated Hospital of Jiangsu University, Zhenjiang, Jiangsu, China; 3 The Central Hospital of Xiaogan, Xiaogan, Hubei, China; Public Library of Science, UNITED KINGDOM

## Abstract

**Background:**

Striae gravidarum is a common skin problem of considerable cosmetic concern for many pregnant women. Various risk factors associated with the development of striae have been reported, with conflicting results.

**Objectives:**

To analyze the risk factors of striae gravidarum in Chinese primiparous women and to provide evidence relevant to the prevention of this condition.

**Methods:**

Singleton primiparous pregnant women who were hospitalized for delivery were included, and relevant data were collected. Independent risk factors associated with striae gravidarum in women with and those without striae gravidarum were identified using logistic regression.

**Results:**

Among 213 singleton primiparous pregnant women, 125 had striae gravidarum, yielding a prevalence of up to 58.9%. There was a significant variation (*P* < 0.05, odds ratio >1) between the striae gravidarum and non-striae gravidarum groups in terms of several factors, including younger maternal age, weight gain during pregnancy, body mass index gain, uterine height, abdominal girth, and positive family history. Factors such as maternal height, birth weight, systemic disease, skin type, and neonatal gender did not significantly differ between the 2 groups.

**Conclusion:**

This study showed that the independent risk factors for striae gravidarum in primiparous women were younger maternal age, weight gain during pregnancy, body mass index gain, uterine height, abdominal girth, and a positive family history. The severity of striae gravidarum was associated with weight gain during pregnancy, body mass index gain, abdominal girth, and timing of the onset of striae gravidarum.

## Introduction

Striae gravidarum (SG), or “striae marks,” is a common cosmetic nuisance that occurs in pregnant women. Although it is not considered medically dangerous to the mother or the fetus, SG can engender a considerable psychological burden in affected women. Moreover, it was reported that women who developed striae were at a higher risk for pelvic organ prolapse [1]. SG frequently appears in the second or third trimester of pregnancy, typically on the abdomen and breasts and less commonly on the buttocks, hips, and thighs [2]. Initially, the lesions are pink to violaceous and may be edematous. Over months to years, the lesions mature into white, shiny, atrophic, and crinkly streaks that are permanent [3]. The prevalence of SG is dependent on the population studied. The estimated prevalence in the general population ranges from 50% to 90% [4], making it one of the most common skin problems during pregnancy. Nevertheless, the etiological mechanisms and true cause of SG remain unknown. It has been reported that relaxin, estrogen, and adrenocortical hormones may have a role in the development of striae, given their effects on collagenous tissue [5]. However, the key biological driver underlying this change remains unknown. Many risk factors associated with striae have been studied among different populations in recent years, but the findings have not been consistent. The proposed risk factors for the development of SG include a positive family history [4], weight gain during pregnancy [6], maternal age [7], and neonatal birth weight [7]. Other factors have also been investigated, including impaired glucose tolerance [8,9], hair color [5,9], nutrition [5,10], and the gender of the neonate [9]. However, no data are available about the risk factors for the development of SG in the Chinese population.

The aim of our study was to determine the prevalence of SG in Chinese primiparous women and identify the risk factors associated with its development.

## Methods

To develop the questionnaire used in our survey, a detailed literature review was conducted and potential risk factors for SG were collected. Considering the features of the Chinese population, some factors were not included, such as skin color and eye color. Finally, a total of 16 variables were determined and all of them were reported in more than 1 study, including maternal age [4,7,11–15], height [15,16], weight gain during pregnancy [4,6,7,11,14,15], body mass index gain during pregnancy [4,7,11–15], smoking [11,15] and alcohol intake history [11,13], family history of striae [4,7,11,13–15], use of anti-striae products [11], week of onset of SG [4,7,11], skin type [7,11,15], number and color of the striae [7], uterine height and abdominal girth before delivery [8,11,15], birth weight [7,11,13–15], and neonatal gender [7,11,15].

Two scales were used in our questionnaire: the Fitzpatrick-Pathak classification [17] and the Atwal scoring system [7]. The Fitzpatrick-Pathak classification is used for skin type evaluation, and the Chinese version of this scale has been widely used in China[18–22]. This scale has been shown to be a valid instrument for the evaluation of skin type in the Chinese female population, and there is a certain correlation between the Fitzpatrick-Pathak skin type and the minimal erythema dose as well as the minimal persistent pigmentation dose, which are 2 objective parameters that reflect skin sensitivity to ultraviolet radiation [23]. The other scale, the Atwal scoring system, was used to evaluate the severity of striae. We translated the original version of the scale to create the Chinese version and then performed backtranslation. As there is no standardized instrument for evaluating the overall severity of striae for the patients, 3 experienced dermatologists were invited to evaluate the validity of the contents. This scale covers 4 common sites of striae development, and includes the number and degree of coloration of the striae. The raters reached an agreement that this scale could be used to evaluate the overall severity of striae and that the included items were sufficient. Finally, 2 investigators carried out a pre-survey by using this scale in a small number of pregnant women, and found that this instrument could meet our initial requirement and expectation. The results of the pre-survey were also comparable to those of previous studies.

Study approval was obtained from the biomedical research ethics review committee of Jiangsu University Hospital, and written informed consent was obtained from all study participants. Singleton primiparous pregnant women (≥37 weeks) underwent investigations at the Affiliated Hospital of Jiangsu University between July and December 2015. Pregnant women who were at full term and nulliparous women with singleton deliveries met the inclusion criteria. Cases of multiple pregnancy, premature birth, fetal death, and systemic diseases (such as diabetes mellitus, hypertension, hyperthyroidism, or connective tissue diseases) and those involving use of drugs were excluded from the study.

The data of a previous study [7] that was conducted under the same working conditions were analyzed using PASS software, which revealed that a total of 125 cases would be required at a power of 90% and a significance level of 0.05. According to our pre-survey, the prevalence of SG is around 56% and a total sample number of 223 was determined.

The data of all variables except 2 were collected via the questionnaire survey and physical examination, whereas neonatal birth weight and gender were recorded after birth. For striae evaluation, 2 investigators (Jianling Huang and Ying Wang) independently examined each participant, and any differences in opinion were resolved through a discussion. To assess the severity of the striae, the number of striae and the degree of erythema at 4 common sites (abdomen, hips, breasts, and thighs/buttocks) were evaluated using the Atwal scoring system. Striae were scored up to a maximum of 6 points at each site: 0–3 for the number of striae present and also 0–3 for the degree of erythema. The number of striae was recorded as follows: no striae, 0; <5 striae, 1; 5–10 striae, 2; >10 striae, 3. The degree of erythema was recorded as follows: no erythema, 0; mild (light red or pink) erythema, 1; marked (dark red) erythema, 2; and violaceous (purple) erythema, 3. The scores were then totaled out of a maximum of 24, and the participants were divided into the following 4 groups: 0–3, “none” or “no” significant striae; 4–9, “mild” striae; 10–15, “moderate” striae; and >16, “severe” striae. Silvery white striae were considered to be old striae that developed before pregnancy and were therefore not included in the analysis.

Primiparous women were divided into 2 groups according to the presence or absence of SG, and certain characteristics were compared. In addition, we assessed the severity of the striae and also determined whether the severity was related to certain variables. The data were analyzed using SPSS version 17.0. Univariate analysis was performed based on Student’s *t* test for continuous factors and either Pearson’s χ^2^ test or Fisher’s exact test for categorical factors. A multivariate logistic regression was performed to select factors independently associated with the occurrence of SG. The significance level was set at 5%.

## Results

In this survey, questionnaires were received from 229 singleton primiparous pregnant women, 213 of which were filled out completely. Of the 213 women enrolled in the study, 125 (58.9%) developed SG. Among these women, SG appeared at different sites including the abdomen (111, 88.8%), hips (36, 28.8%), thighs (35, 28%), and breasts (8, 6.4%). According to the Atwal scoring system, the severity of the striae was as follows: 76.8% (96 of 125) were graded as mild, 17.6% (22 of 125) were moderate, and 5.6% (7 of 125) were severe. In terms of the striae onset time, SG appeared most commonly between 24 and 35 weeks of pregnancy. The mean age of the SG group (26.39 ± 2.75 years) was lower than that of the non-SG group (27.84 ± 3.67 years). In addition, there were significant differences in several factors, such as weight gain, body mass index gain during pregnancy, uterine height, and abdominal girth, between the 2 groups (*P* < 0.05). However, no statistically significant differences were observed between the 2 groups with regard to maternal height and neonatal weight (Table 1). Of the 125 women who developed SG, 40% (50 of 125) reported a family history of SG, whereas 38.4% (48 of 125) did not. The remaining women with SG did not know whether their sisters or mothers had striae during pregnancy. No significant difference was observed between the 2 groups in terms of other chronic diseases, skin type, and neonatal gender (Table 2).

**Table 1:** Characteristics of women with and without striae gravidarum (continuous factors).

	SG (n = 125)	Non-SG (n = 88)	*t*	*P*-value
Age (y)	26.39 ± 2.75	27.84 ± 3.67	-3.293	0.001^a^
Height (m)	1.61 ± 0.04	1.62 ± 0.05	-1.532	0.127
Weight gain during pregnancy (kg)	16.46 ± 4.57	14.06 ± 4.18	3.912	0.004^a^
BMI^b^ gain during pregnancy	6.35 ± 1.78	5.36 ± 1.56	4.202	0.000^a^
Birth weight (kg)	3.37 ± 0.43	3.27 ± 0.37	1.684	0.094
Uterine height (cm)	35.02 ± 2.46	34.02 ± 2.86	2.708	0.007^a^
Abdominal girth (cm)	102.71 ± 5.65	99.45 ± 6.62	3.858	0.000^a^

Data are presented as mean ± standard deviation. SG, striae gravidarum; non-SG, without striae gravidarum; BMI, body mass index.

^a^Statistically significant, *P* < 0.05.

^b^BMI = weight (kg) / height^2^ (m^2^).

**Table 2:** Characteristics of women with and without striae gravidarum (categorical factors).

	SG, n (%)	Non-SG, n (%)	*X*^*2*^	*P*-value
Family history			62.618	0.000^a^
No	48 (38.4)	81 (92.0)		
Yes	50 (40.0)	6 (6.8)		
Unknown	27 (21.6)	1 (1.1)		
Other diseases			0.002	0.967
No	112 (89.6)	79 (89.7)		
Yes	13 (10.4)	9 (10.2)		
Skin type			2.501	0.475
I/II	40 (32)	24 (27.3)		
III	62 (49.6)	44 (50)		
IV	23 (18.4)	20 (22.7)		
Neonatal gender			1.395	0.238
Boy	60 (46.4)	35 (39.8)		
Girl	65 (51.2)	53 (60.2)		

SG, striae gravidarum; non-SG, without striae gravidarum.

^a^Statistically significant, *P* < 0.05.

Logistic regression analysis showed that the independent risk factors for SG in primiparous women were younger maternal age, weight gain during pregnancy, body mass index gain, uterine height, abdominal girth, positive family history, and use of anti-striae products. The severity of SG was associated with weight gain during pregnancy, body mass index gain, abdominal girth, and earliest time of SG onset (Table 3).

**Table 3:** Risk factors for SG in multiple logistic regression analysis.

Factors	B	SE	Wald	*P*-value	OR	95% CI
Age (y)	-0.145	0.047	9.690	0.002^a^	0.865	(0.790–0.948)
Weight gain during pregnancy (kg)	0.125	0.034	13.591	0.000^a^	1.133	(1.060–1.212)
BMI gain during pregnancy	0.349	0.089	15.334	0.000^a^	1.418	(1.190–1.688)
Uterine height (cm)	0.158	0.060	6.876	0.009^a^	1.717	(1.041–1.317)
Abdominal girth (cm)	0.090	0.025	13.177	0.000^a^	1.094	(1.042–1.148)
Family history	2.144	0.392	29.974	0.000^a^	8.531	(3.960–18.377)
Usage of anti-striae products	1.411	0.303	21.648	0.000^a^	4.101	(2.263–7.430)

SE, standard error; OR, odds ratio; CI, confidence interval; BMI, body mass index.

^a^Statistically significant, *P* < 0.05.

In addition, we focused on the degree of severity based on selected characteristics. According to the Atwal scoring system, we graded striae as mild, moderate, or severe. We divided the women into 2 groups based on whether they had mild or moderate/severe SG. Moderate/severe SG was defined as an Atwal score of ≥10. Finally, we found that weight gain during pregnancy, body mass index gain, and abdominal girth were higher in women with moderate/severe SG than in those with mild SG (*P* < 0.05). Moreover, the time point of the occurrence of SG was earlier in the moderate/severe group than in the mild group. However, no significant differences were observed between the 2 groups in terms of maternal age and uterine height (Table 4).

**Table 4:** Degree of severity based on selected maternal characteristics.

	Moderate/severe SG^a^		*P*-value
	Yes (n = 96)	No (n = 29)	
Age (y)	26.21 ± 2.48	26.45 ± 2.84	0.681
Weight gain during pregnancy (kg)	18.58 ± 4.05	15.82 ± 4.54	0.004^b^
BMI gain during pregnancy	7.13 ± 1.63	6.12 ± 1.79	0.003^b^
Uterine height (cm)	35.69 ± 2.69	34.81 ± 2.37	0.093
Abdominal girth (cm)	105.86 ± 5.43	101.76 ± 5.39	0.000^b^
Earliest week of SG appearance	27.0 ± 5.21	31.01 ± 4.26	0.000^b^

SG, striae gravidarum; BMI, body mass index.

^a^Moderate/severe SG means Atwal score ≥10.

^b^Statistically significant, *P* < 0.05.

## Discussion

To our knowledge, this is the first study to explore the risk factors associated with SG in the Chinese female population. The prevalence of SG was 58.9%, which is consistent with the prevalence in the general population (50–90%) [4]. This prevalence was higher than that reported for white British women (42%) [7], but lower than the prevalence reported in Australia (60%) [8] and Turkey (75.4%) [11].

Similar to the studies of Ersoy et al. [11] and Thomas and Liston [12], we also observed that younger pregnant women were more likely to develop SG. Most likely, this is due to the increased fragility of fibrillin in younger women [24]. In this study, weight gain and body mass index gain during pregnancy were risk factors for the development of SG. The results were consistent with some studies [7,13] but different from others [4,16]. Therefore, it is necessary to investigate different populations to confirm the risk factors. Considering that mechanical stretching of the skin can damage fibrillin and has a role in the pathogenesis of SG [7], we included uterine height and abdominal girth in our investigation. Uterine height and abdominal girth were higher in the SG group than in the non-SG group. In addition, among the variables evaluated in our study, a positive family history was the most significant factor for the occurrence of SG, which is similar to the results of the study by Kasielska-Trijan et al. [14], and suggests that genetic factors may play a key role in SG. Some recent studies [25–27] investigated the effect of genetic factors in the mechanism of SG. The expression of genes involved in fibroblast metabolism, such as those coding for collagen, elastin, and fibronectin [25], was lower in striae distensae skin than in normal skin. SG was also described in monozygotic twins [26]. Overall, the role of genetic factors in the development of SG warrants consideration.

In our survey, skin type was not a significant factor in the occurrence of SG, which is consistent with a previous study [15]. This finding can be attributed to the fact that a large proportion of the women in our population had Fitzpatrick type III skin. Although Atwal et al. [7] reported that neonatal gender and weight were significantly associated with the development of SG, we observed no significant differences in these factors between the 2 groups in the present study. The reason is unknown and must be further investigated.

We analyzed the severity of SG based on specific characteristics, and found that 4 factors seemed to affect its severity: weight gain during pregnancy, body mass index gain, abdominal girth, and the earliest timing of SG onset. These factors may all contribute to increased skin tension and damage the tissue structure of the skin.

In addition to the above variables, other factors such as smoking history, drinking history, and attempts to prevent SG are not presented in the tables. The proportions of smokers and drinkers in our study were too small to demonstrate any difference with regard to the development of SG. In recent years, some new risk factors have been assessed in studies on this condition. Narin et al. [28] investigated the effect of geography and altitude on the formation of SG, and found that striae formation was significantly more common in more highly elevated areas. Interestingly, some researchers sought to explore the relationship between striae and other diseases such as polycystic ovarian syndrome and acne gravidarum [29].

Interestingly, the use of anti-striae products seemed to increase the likelihood of striae, and we assumed that it was because many women used anti-striae cream or oil after the occurrence of SG. In future studies, the timing of the commencement of use of such products must be clarified. According to the results of a Cochrane meta-analysis, all treatment strategies were found to be minimally effective in the prevention of SG [11].

Some limitations in our study should be noted. For example, only primiparous women in Zhenjiang City were included, and the evaluated variables were also limited. However, as no related data from the Chinese population have been published as yet, further insights will be achieved by comparing the results among different members of the population. In future studies, other variables such as education and economic levels, intrinsic genetic factors, and adolescent history of striae should be included.

In conclusion, in this study, the development of SG was associated with younger maternal age, weight gain during pregnancy, body mass index gain, uterine height, abdominal girth, and family history. Given that family history may be the most significant factor in the development of SG, further research should focus on the relationship between genetic factors and SG.
